# Evaluation of an online training program in eating disorders for health professionals in Australia

**DOI:** 10.1186/s40337-015-0078-7

**Published:** 2015-11-06

**Authors:** Rachel S. Brownlow, Sarah Maguire, Adrienne O’Dell, Catia Dias-da-Costa, Stephen Touyz, Janice Russell

**Affiliations:** The University of Sydney, Sydney, Australia; The Centre for Eating and Dieting Disorders, Sydney, NSW Australia

**Keywords:** Eating disorders, Online training (e-learning), Health professionals, Identification, Treatment

## Abstract

**Background:**

Early detection and treatment of eating disorders is instrumental in positive health outcomes for this serious public health concern. As such, workforce development in screening, diagnosis and early treatment of eating disorders is needed. Research has demonstrated both high rates of failure to accurately diagnose and treat cases early and low levels of perceived access to training in eating disorders by health professionals–representing an urgent need for clinician training in this area. However, significant barriers to the access of evidence-based training programs exist, including availability, cost and time, particularly when large geographic distances are involved. Online learning presents a solution to workforce challenges, as it can be delivered anywhere, at a fraction of the cost of traditional training, timing is user controlled, and a growing body of research is demonstrating it as effective as face-to-face training. The Centre for Eating and Dieting Disorders in Australia has developed an Online Training Program In Eating Disorders, to educate health professionals in the nature, identification, assessment and management of eating disorders. The aim of the current study was to evaluate the ability of this online learning course to improve clinician levels of knowledge, skill and confidence to treat eating disorders. As well as its effect on stigmatised beliefs about eating disorders known to effect treatment delivery.

**Methods:**

One-hundred-eighty-seven health professionals participated in the program. A pre training questionnaire and a post training evaluation examined participants’ levels of knowledge, skill and confidence to treat eating disorders, as well attitudes and beliefs about people with eating disorders.

**Results:**

Significant improvements in knowledge, skill, and confidence to treat eating disorders was found between pre and post program assessment in health professionals who completed the course, along with a significant decrease in stigmatised beliefs about eating disorders.

**Discussion:**

The results of this study demonstrated that the online training program was an effective tool in increasing health professionals’ level of knowledge, skill and confidence to treat people with eating disorders. The results also demonstrated that online training reduced health professionals’ personal bias towards people with eating disorders. Limitations of this study include the use of self-report measures rather than observation of the health professional in clinical practice. As a result, it is not possible to make determinations regarding the translation of these results to clinical settings.

**Conclusions:**

The findings of this study suggest that online training programs may present an innovative solution to the considerable workforce development challenges faced by clinicians needing training in eating disorders.

## Background

Eating disorders (EDs) are biologically based serious mental illnesses [1] which multiple medical and psychological sequelae [2]. The impact of an ED is often on young women as they enter adolescence and young adulthood, with considerable implications for their immediate and long-term development. As such they represent a significant cost for the individual, their family members and society [3]. EDs are the leading cause of disability due to mental health disorder amongst females aged from 10 to 24 years [4]. Mortality rates are substantially higher than in the wider population, and higher than for any other psychiatric illness [5]. Two recent studies [6, 7] have shown suicide contributes significantly to the mortality rate and there are high levels of self-harm evident in this illness group [8]. Comorbidity is the norm rather than the exception, with anxiety (>50 %), depressive disorders (20–80 %) and substance abuse all common and adding significantly to the overall burden of disease [9–11]. The research literature suggests that eating disorders occur at similar rates across Western countries. For example, in the Australian community, lifetime prevalence of an eating disorder is as high as 15 % in females and 3 % in males [12]. The estimated financial cost in Australia associated with disability-adjusted life years attributable to eating disorders is greater than that for anxiety and depression combined [2], rendering it a significant public health concern.

Early diagnosis and treatment are essential to a good outcome, but barriers presently exist across the health system. Even though the spectrum of health professionals dealing with EDs has increased in recent years, specific training in or experience with ED patients is still lacking [13]. In their early stages, before physical symptoms arise, psychological symptoms of an eating disorder can be well hidden. Patients may present with other complaints such as fatigue, abdominal pain and constipation, so that up to 50 % of cases can go undetected in the primary care setting [14]. In the emergency department the picture is similar, lack of training combined with atypical presentations has been demonstrated to result in the majority of eating disorders remaining undiagnosed for some years [15]. Another study of family physicians in the UK [16] found that females were more likely to receive a diagnosis than males, even when symptoms were identical, and overall there was a reluctance to diagnose AN, even in patients with very low weight status. The authors also concluded that there were major discrepancies between primary care decision-making and recommended guidelines for ED patients. A study in UK pediatric hospitals [17] to determine the training needs of doctors found a large proportion (73 %) perceived eating disorders to be of high importance, but rated knowledge, skill base and confidence to treat as low. A national US survey [18] found a lack of essential treatment skills for eating disorders in resident doctors and an associated reduction in empathy for these patients.

Lack of empathy combined with negative stereotypical images of patients with eating disorders and their families are not uncommon [19]. Stigmatisation remains a significant barrier to receiving effective treatment. One study [20] found elevated levels of stigmatisation towards patients with AN based on beliefs about personal blame for the illness, and that these were higher than for obese patients. Another study investigating stigmatisation [21] reported perceptions that AN was caused by poor parenting and a lack of self-discipline with little involvement of biological factors, and perhaps most importantly that stigma-induced shame caused by these perceptions discouraged AN sufferers from seeking treatment.

In Australia, and elsewhere in the world, workforce education poses a particular set of problems. Australia has a generalist mental health model, so that care for EDs is delivered for the most part by clinicians in general practice, community clinics, general medicine, emergency departments and psychiatric wards. Funding for specialist services is limited, which means that training of health professionals at all places where contact with individuals with EDs arise becomes a priority if adequate evidence-based care is to be provided. Further, there is a tyranny of distance with communities being spread across large geographic areas, with relatively few metropolitan hubs of expertise. Like many countries, most health infrastructure in Australia is located in large urban centres, leaving rural and regional areas under-serviced. This means that many health care professionals working in regional and rural locations, and the clients with eating disorders that present to them, are disadvantaged in terms of access high quality care and training.

Online training presents a potential solution as it can be delivered anywhere, at any time, and at a fraction of the costs of traditional training methods. There is growing body of research [22, 23] suggesting that internet-based medical education is just as effective as the more traditional forms. Internet-based training revealed equivalent effectiveness in imparting knowledge [22, 24, 25], with similar–and sometimes greater–levels of satisfaction [26] than traditional learning, and can produce changes in behaviour and sustained knowledge gains over time [27]. A systematic review and meta-analysis of internet-based learning modules, identified certain basic principles such as interactivity, practice exercises, repetition and feedback to the user as being most important to ensure user satisfaction and to achieve real learning outcomes [28].

While several internet-based programs targeting self-help and support in eating disorders [29–33], exist there are few reported in the literature that target health professionals. A Canadian group [34] evaluated a web-based ED prevention program designed for school teachers and health practitioners, orientated around the factors that influence body image and dieting and the inherent risk of disordered eating patterns. They found the program increased both knowledge and efficacy in the cohort.

Recently Fairburn and Wilson [35] have argued that evidence based therapies are poorly propagated across the health system and as such “internet-enhanced training” [36], is required to disseminate evidence based training across the health workforce. Support for using internet-based approaches to solve the issues of workforce training in eating disorders has now earnt a wider audience [37].

To address the issues of workforce competencies and skill to detect, diagnose and manage eating disorders in the Australian health workforce, The Centre for Eating and Dieting Disorders (CEDD) has developed an online learning program for health professionals in the nature, identification, assessment and management of eating disorders. This comprehensive, interactive core competencies based training is the first of its kind internationally, the result of a collaborative project spanning a number of care settings and institutions, and draws on the extensive clinical and research experience of internationally renowned specialists in the field. This interactive program provides comprehensive training in diagnosis along with the medical, psychological and dietetic management of these mental illnesses.

The current study examined the impact of the above program on the knowledge, skills, willingness, confidence and stigma levels of health professionals treating eating disorders within the Australian health workforce.

## Method

### Participants

The study was reviewed and approved by the Royal Prince Alfred Hospital. The program was advertised globally via online advertisements on mental health related institute websites and the CEDD website and email list serve. Health professionals from Australia and other countries (Indonesia, United Kingdom and USA) provided informed consent to participate in the study and had 6 months to complete the online training program. Health professionals registered progressively from July 2012 to October 2013. During this 6-month period, 187 health professionals completed the program.

### Procedure

#### Training program

Training modules and topics were designed and written by both expert and novice panels of health professionals inclusive of nurses, dietitians, psychologists, psychiatrists and general practitioners (GPs), and curriculum development was based on literature reviews and expert consensus building. The program comprises five modules–understanding eating disorders, diagnosis, preparation for treatment, treatment and management–each 3.5 h in length, and combines text-based psycho-education, role-plays, interactive exercises and quizzes, as well as video footage of sufferers and their families. Each of the five learning modules contains a core curriculum, required to pass the quiz, an ‘in practice’ section with additional clinical tools and role-plays, and a resources section with key readings and resources.

#### Pre and post questionnaires

Program participants were required to complete a pre training questionnaire prior and a post training evaluation upon completion of the program. Pre and post training self-report questionnaires were developed by the authors and are available over the Internet for the purpose of evaluating the effectiveness of the online training program. Participants were asked 10 questions rating their current level of knowledge (e.g. the risk factors associated with the development of an ED), and 9 questions rating their skill level for assessment and treatment of EDs (e.g. ability to conduct ED screening) on a 5-point Likert scale (1. Very low; 2. Low; 3. Moderate; 4. High; 5. Very High). Participants were asked 6 questions regarding their personal attitudes and beliefs about people with an ED to assess stigma (e.g. they are to blame for their condition) and asked to rate on a 5-point Likert scale (1. Strongly Disagree; 2. Disagree; 3. Neutral; 4. Agree; 5. Strongly agree). Participants rated their willingness and confidence in treating each of the eating disorder subtypes on a 5-point Likert scale ranging from 1 (Not at all willing/Not at all confident) to 5 (WillingIncrease in current knowledge of eating disorders and skill level[Para, ID = Par21]/Confident). Additional items assessing demographic data including gender, age, primary professional discipline, work setting, length of time in practice and prior experience treating EDs were included at baseline. Participants also rated their experience of the online training program.

### Data analyses

Data were analysed using SPSS Version 20.0. Wilcoxon signed ranked test was used to investigate changes between pre and post training evaluation scores for items and subscales (knowledge, skill and bias) as appropriate for nonparametric data.

## Results

The mean age bracket of the 187 participants was 31 to 40 years and the vast majority were females (91.4 %). All of these participants completed the program in its entirety, and all of the questionnaires at pre and post. Half of the participants (49.2 %) reported having been in practice for less than 5 years. Of those who completed the online learning program, 34.6 % were psychologists, 22.7 % nurses, 19.5 % dietitians and 9.2 % social workers. The majority of respondents indicated they worked in a community health or mental health centre (40.2 %). More than half of the respondents (62.2 %) indicated their employment setting as being within the metropolitan area and the other 36.8 % worked in a rural/regional employment setting.

Almost half (46.0 %) of the respondents reported they don’t have the skills to treat ED patients. In addition, 34.0 % of respondents indicated that there were not enough resources available to help them adequately treat these patients with only 2.7 % indicated they did not like treating this patient group or that it was too time consuming.

### Increase in current knowledge of eating disorders and skill level

Upon completion of the online training program, participants reported a significant increase in their knowledge about EDs (z = 11.58, *p* < 0.001), and their clinical skills assessing and treating eating disorders (z = 11.42, *p* < 0.001) (see Table 1).

**Table 1:** Changes from pre to post evaluations on knowledge, skill level, stigma and attitudes

Subscale	Pre-Mean (SD)	Post-Mean (SD)	Z score
Knowledge (10 items)	2.64 (0.70)	3.74 (0.53)	11.58**
Skill level (9 items)	2.68 (0.72)	3.63 (0.60)	11.42**
Stigma and attitudes (6 items)	1.67 (0.52)	1.46 (0.49)	5.76**

** Wilcoxon signed rank test highly statistically significant (*p*-value <0.001)

### Decrease in personal bias toward eating disorders

In addition, upon completion of the online program, participants also endorsed significantly less stigmatised beliefs towards EDs (z = 5.76, *p* < 0.001) (see Table 1).

### Increase in confidence to treat eating disorders

Health professionals reported a significant increase in their confidence to treat each EDs after completing the online training program, across all ED subtypes, AN (z = 9.12, *p* < 0.001), BN (z = 9.85, *p* < 0.001), BED (z = 9.85, *p* < 0.001) and EDNOS (z = 9.55, *p* < 0.001).

### Willingness to treat eating disorders

Participant’s willingness to treat BED (z = 2.58, *p* = 0.01) and EDNOS (z = 2.57, *p* = 0.01) increased significantly after completing the online training program. There was no difference between pre and post training evaluations in health professionals’ willingness to treat anorexia nervosa (z = 0.53, *p* = 0.60), and bulimia nervosa (z = 1.74, *p* = 0.08).

### Online training program evaluations

The acceptability of the program Overall the program received overwhelmingly positive self-report reviews. 96.2 % of respondents indicated that their current clinical practice had improved as a result of completing the online learning program. 98.4 % of respondents indicated that their expectations of the online learning program were met. 99.5 % of respondents indicated that the program was relevant or highly relevant to their practice. 95.8 % of respondents indicated that the program met their needs. One hundred percent of respondents stated that they would recommend the online learning program to other health professionals.

## Discussion

The purpose of the current study was to evaluate the effectiveness of an online training program specifically developed to disseminate evidence-based knowledge to health professionals working in the area of EDs. Online training programs offer a significant advantage over face-to-face training, particularly when considered within the context of rural and remote geographic locations, such as those within Australia, where training programs may not be easily accessed.

The results of this study demonstrated that the online training program was an effective tool in increasing health professionals’ level of knowledge, skill and confidence to treat people with EDs. The results also demonstrated that online training reduced health professionals’ personal bias towards people with EDs.

The lack of significant change on some measures of the willingness to treat item was likely result of a ceiling effect–in that pre willingness to treat was in this cohort very high.

Limitations of this study include the use of self-report measures rather than observation of the health professional in clinical practice. As a result, it is not possible to make determinations regarding the translation of these results to clinical settings. Another limitation included the absence of a wait list or manual control condition in order to compare different training methodologies.

The importance of this study lies firstly in the uniqueness of its subject, which targeted a broad range of health professionals, participants including psychologists, nurses, dietitians and social workers. The comprehensive format allowed access to best practice strategies from experts in the ED field covering a wide range of medical, dietetic and psychological management practices. Despite the diverse backgrounds of the participants the results were positive in all areas of assessment.

We note that no GPs were involved in the evaluation of this program and hence we are unable to report on the effect of the program for this important health discipline. This is believed to be a result of advertising and promotion of the program being more effective within the public hospital and clinic system, and GPs being a difficult sub-population to engage in learning both due to access to them, and considerable professional time constraints. Future research needs to evaluate the effectiveness of the program for GPs.

To our knowledge this online training program is the first of its kind internationally. The results of the study suggest that it may be an effective tool for the dissemination of knowledge, skill and confidence to general health clinicians in the diagnosis, treatment and ongoing management of eating disorders.

## Conclusions

This study demonstrates support for a novel avenue of training in the area of EDs. Based on the results of this study, it is possible to conclude that this online training program offers the potential to make a substantial contribution to the development of the ED workforce. The online program has the capacity to reach large numbers of health care professionals and the ability to overcome barriers to access including time, cost and distance. Despite improvements in treatments for EDs, it is apparent that there are unmet needs for treatment and barriers to treatment-seeking amongst individuals with EDs, and a lack of workforce training is one of the factors contributing to this. These barriers included stigmatising attitudes and a lack of ED specific skills amongst health professionals. This online training program was able to increase knowledge, skill and confidence in health care professionals and also reduced personal bias towards sufferers.
