# Erratum to: Evaluation of an online training program in eating disorders for health professionals in Australia

**DOI:** 10.1186/s40337-016-0097-z

**Published:** 2016-03-10

**Authors:** Rachel S. Brownlow, Sarah Maguire, Adrienne O’Dell, Catia Dias-da-Costa, Stephen Touyz, Janice Russell

**Affiliations:** The University of Sydney, Sydney, Australia; The Centre for Eating and Dieting Disorders, Sydney, NSW Australia

## Erratum

Unfortunately, the original version of this article [1] contained an error. After publication it was noticed the “Acknowledgements” section was missing. This should have read, “Many health professionals contributed to the development of this online learning program, we thank them all for their contribution and expertise in particular Jeremy Freeman, Peta Marks and Mel Hart.”
